# Reviewers in 2019

**DOI:** 10.1098/rsos.200403

**Published:** 2020-03-25

**Authors:** Jeremy KM Sanders

**Affiliations:** Editor-in-Chief

The editors of *Royal Society Open Science* would like to thank all reviewers who contributed their time and expertise by refereeing manuscripts submitted throughout 2019. From previous feedback, we know that most reviewers value recognition of their efforts by the journal and through services such as *Publons* which are integrated with *Royal Society Open Science*.

To provide an opportunity for recognition, we list the names of all reviewers (unless they have opted out). This article is made permanent and citeable by its digital object identifier (DOI). We encourage you to quote your contribution in your applications for tenure, promotion and grants, or other forms of assessment. Where you have provided it, we have included your ORCID, so your contribution can be unambiguously assigned to you.

The team greatly appreciate all the work our reviewers do on behalf of the journal, and we look forward to working with you again in 2020.

Estes A
Huang J
Nittono H
Romano D
Sah P
Vanzela LM
Abarra I
Abdallah S
Abdelrahman E
Abdessemed A https://orcid.org/0000-0001-5190-5991
Abdey J
Abobakr A
Aboelalla M https://orcid.org/0000-0002-8579-0093
Abubakar AM
Acar I
Adams M
Adams R https://orcid.org/0000-0002-8053-0671
Adzhubai A
Aerts P https://orcid.org/0000-0002-6867-5421
Agarwal V
Aguilar-Vega M
Ahmed I http://orcid.org/0000-0003-3868-2919
Ahmed MR
Åkermark B
Akhter A https://orcid.org/0000-0001-6482-2116
Akinyoola J
Akizuki S
Akman M
Al Rawi A
Alarcón J
Alarcon E
Albert C
Albertini M
Alberts B
Alderink G
Alessandretti L
Ali A
Ali I
Ali S
Ali S
Al-Kheetan M https://orcid.org/0000-0001-8366-7932
Allan J
Allen P
Allen S
Almehizia A https://orcid.org/0000-0001-8711-3873
Almeida J
Almeling L
Alsultan A https://orcid.org/0000-0001-8807-7587
Altmann E https://orcid.org/0000-0002-1932-3710
Alvarez E
Alves H
Alves R
Amador-Vargas S
Ambade A
Amiri R
An J
An Q
An R
Ana M
Andrada E http://orcid.org/0000-0002-9300-4056
Anfinogentov S
Angione C https://orcid.org/0000-0002-3140-7909
Anglés-Cano E https://orcid.org/0000-0001-7891-3975
Anjanapura R
Anshits A
Anton S
Antonioni A https://orcid.org/0000-0002-5788-3348
Ao Y
Arcibar Orozco JA
Ardeshirylajimi A
Ares AE
Arora J
Asensio E
Ashcraft M https://orcid.org/0000-0003-0863-7666
Assogbadjo A
Ata MS
Augusto M
Aung T
Avgar T
Azhagapillai P
Bacon J
Badaan V
Bae JW
Baert J
Bagheri H
Bai X https://orcid.org/0000-0003-3761-0526
Bai Y
Baino F
Bajda T https://orcid.org/0000-0003-3849-5558
Baker C
Ballard G
Ballesta A
Banerjee J
Banerjee T
Bao S https://orcid.org/0000-0003-1295-3389
Bapst D
Barber-Meyer S
Barclay P http://orcid.org/0000-0002-7905-9069
Bardi L
Barghash A
Barma SD
Barnes R
Barnett J https://orcid.org/0000-0001-9789-4132
Barr D
Barrio P
Barry O
Barta Z
Bartolini Lucenti S https://orcid.org/0000-0003-1280-5378
Barton J
Barton M
Barts N
Bastakoti B
Bastian B https://orcid.org/0000-0003-4619-3322
Bastos A
Baumann-Pickering S
Baumard N
Baynes T
Bebber MR
Beck M https://orcid.org/0000-0002-1737-0951
Becker D https://orcid.org/0000-0003-4315-8628
Beedholm K
Beffara-Bret B https://orcid.org/0000-0002-0586-6650
Begeer S
Begus K https://orcid.org/0000-0002-0264-4534
Beißert H
Belal F
Bellas E
Bennetts R
Bentley B
Benton M https://orcid.org/0000-0002-4323-1824
Bentz C https://orcid.org/0000-0001-6570-9326
Berggren N
Berli F
Berlow M
Berwick B
Beutel R
Bezci S https://orcid.org/0000-0002-7237-6635
Bharadwaj V https://orcid.org/0000-0001-5851-9807
Bhaumik A
Bhunia S https://orcid.org/0000-0003-3587-3509
Bianucci G https://orcid.org/0000-0001-7105-0863
Bieleke M
Bierbach D
Billingham J https://orcid.org/0000-0002-4392-5770
Binda P
Birn-Jeffery A
Bishop T
Blank H
Blasius B
Blinch J
Bliss-Moreau E https://orcid.org/0000-0002-0740-5612
Bloom DD
Bloom S
Bodawatta K https://orcid.org/0000-0002-6095-9059
Bode N https://orcid.org/0000-0003-0958-5191
Boisgontier M
Bókony V
Bomphrey R
Bonduà S
Bonnaud-Ponticelli L https://orcid.org/0000-0001-7510-5032
Bonnet T
Bontempi E
Boonekamp J
Bossenbroek J
Botella C
Botero-Castro F https://orcid.org/0000-0002-5062-8272
Botta F https://orcid.org/0000-0002-5681-4535
Bourne D
Bowen T
Bowman R https://orcid.org/0000-0002-1531-8199
Brady O
Brauer F
Brezinsek S
Breznau N
Briga M http://orcid.org/0000-0003-3160-0407
Briggs C
Brima E https://orcid.org/0000-0002-4525-2709
Brink KS
Brinkløv S
Brocki K
Brockmann A https://orcid.org/0000-0003-0201-9656
Broda M
Brookfield J https://orcid.org/0000-0002-0509-0274
Brooks K https://orcid.org/0000-0003-1424-4092
Broomhall A-M
Brown C
Brown G
Brown G
Browne N
Broxmeyer H
Brüggeller P
Bruijn S https://orcid.org/0000-0003-0290-2131
Bruna M https://orcid.org/0000-0001-7829-5652
Bruzzone L
Bryant D
Bryant G
Buatois L https://orcid.org/0000-0001-9523-750X
Buckeridge J
Budar F
Buggs R
Bugnyar T
Buhl-Mortensen L
Burke C
Burmeister S https://orcid.org/0000-0001-5612-692X
Büsse S https://orcid.org/0000-0002-1657-7950
Butler A
Caciagli L
Cade D
Caginalp C https://orcid.org/0000-0002-3105-3952
Cai Y
Caines A
Cakirlar C
Callone E
Calude A
Calvo F
Campos P https://orcid.org/0000-0002-7823-1998
Cano M
Cao J
Cao Q
Cappellini E
Capraro V https://orcid.org/0000-0002-0579-0166
Carapezzi S https://orcid.org/0000-0002-9271-1189
Carballido J https://orcid.org/0000-0003-3227-8034
Carboch J
Cárdenas J
Cardini F
Carey D
Carr T
Carrasco J
Carrera M
Caruntu G
Casado-Gavalda MP
Casagrande S https://orcid.org/0000-0002-4264-8062
Casimiro H
Castaldi E
Caswell B https://orcid.org/0000-0001-8488-0890
Celik E
Cerasuolo M
Cerbino R
Ceulemans R
Chakrabarti S
Chaminade T https://orcid.org/0000-0003-4952-1467
Chandrasekaran S https://orcid.org/0000-0002-8405-5708
Chandravanshi BS
Chandrika N
Chang Z
Chao D
Chao Y http://orcid.org/0000-0002-8488-2690
Chapple D
Checa A https://orcid.org/0000-0001-7873-7545
Chen C-TA
Chen D
Chen G
Chen J https://orcid.org/0000-0002-1277-7891
Chen P
Chen Q
Chen S
Chen T-H
Chen X
Chen X https://orcid.org/0000-0002-9129-2197
Chen Y
Chen Y-C
Chen Z http://orcid.org/0000-0002-0161-0236
Chen Z
Chen Z https://orcid.org/0000-0001-8292-4861
Cheng B https://orcid.org/0000-0002-7989-0557
Cheng J
Cheng K https://orcid.org/0000-0002-4913-2691
Cherubini P
Cheung B
Chimento M https://orcid.org/0000-0001-5697-1701
Chitta R
Chiu W
Chivers P
Choudhury B
Chough Y
Chow S
Christensen R
Christian J
Christodoulides P
Chu CH
Chung MK
Ciaccio E
Cismondi M
Cisneros J
Cizmic M
Clansey A
Clark A
Clark C https://orcid.org/0000-0002-7692-8150
Clark RM
Clarke R
Clauss M https://orcid.org/0000-0003-3841-6207
Cleland T
Clemente C https://orcid.org/0000-0001-8174-3890
Coello Y
Coleman R
Colinet G https://orcid.org/0000-0002-1850-5504
Collareta A https://orcid.org/0000-0002-6513-8882
Collett T
Collins K https://orcid.org/0000-0002-3379-4201
Considine RV
Constantinescu G
Constantino R
Convey P https://orcid.org/0000-0001-8497-9903
Conway K
Cook G
Cooper N
Coppock A
Cordellier M
Cords M
Corker KS https://orcid.org/0000-0002-7971-1678
Corkeron P https://orcid.org/0000-0003-1553-1253
Corlett P
Corral A
Cortés-Avizanda A
Ćosić M
Costas Rodriguez M
Costeur L https://orcid.org/0000-0001-6213-8556
Cotter S https://orcid.org/0000-0001-5974-7393
Counsell A
Coutanche M https://orcid.org/0000-0002-2232-3519
Coutino-Gonzalez E
Cowan D
Cowen L
Cox R
Cox T
Crawford R
Cremer S
Crevecoeur F
Cristea D
Cronk L https://orcid.org/0000-0002-7583-7417
Crowston K
Csapó E
Cuff A https://orcid.org/0000-0001-9509-4297
Cui X https://orcid.org/0000-0001-8464-7773
Cully A
Curkovic M
Curotto E
d'Onofrio A https://orcid.org/0000-0002-2190-272X
Dąbrowska J
Dabrowska M
Dahanukar N
Dai J
Dai L
Dalmaschio CJ
Dammhahn M
Dando P
D'Angelo L
Daniels L
Danos N
Dantzer B https://orcid.org/0000-0002-3058-265X
Dargent F
Das Dr S https://orcid.org/0000-0002-0147-449X
Das G
Davies S https://orcid.org/0000-0003-3890-7372
Davinelli S
Davison A
Davoust L
Dawson P https://orcid.org/0000-0002-5954-4470
Dawson R
de Aguiar M
de Bakker D
de Bekker C http://orcid.org/0000-0002-7325-172X
de Brevern A
de Bruyn N
de Carvalho M
De Clercq D
de Lannoy C-F
De Martino A
De Pedro L
De Silva R
De Silva R https://orcid.org/0000-0003-0955-6366
De Souza JT
De Tomi G
Dearnley P
DeBruine L https://orcid.org/0000-0002-7523-5539
Decety J
Dechaume-Moncharmont F-X
Dehler C
Dekker T
Del Rao JA
Delgado AV
Della Volpe C http://orcid.org/0000-0002-3473-8092
Demarchi B
Denardin C
Densley Tingley D
Denzinger A
Desai A
Dessler A
Destgeer G
Deufel A
Devaud J-M
Di Giminiani R
Di Giulio M
di Virgilio A
Dieci R
Di-Francesco-Maesa D
Dijkerman C
Dimitrov D
Ding Y
Ding Y
Ding Y https://orcid.org/0000-0002-5953-3800
Dingwell J
Diuzheva A https://orcid.org/0000-0003-2090-1634
Dobrovolny H https://orcid.org/0000-0003-3592-6770
Dominy N https://orcid.org/0000-0001-5916-418X
Donald A
Dong M https://orcid.org/0000-0002-2025-2171
Dong W
Dong Y
Dorgan K https://orcid.org/0000-0002-9687-9675
Dosen S
Doughty P
Dragomir M
Drummond A https://orcid.org/0000-0002-2386-072X
Druzinsky R http://orcid.org/0000-0002-1572-1316
Drzewiecka K
Du L
Du D
Du H
Du J
Dubey I
Duboscq J http://orcid.org/0000-0002-6342-6709
Dudek B
Dudek N
Dudek W
Duman O
Dumont B
Dumont M
Dumontheil I
Duncan K
Dvoretsky V
Dykman L
Dzhelyova M
Ebensperger L
Edelaar P https://orcid.org/0000-0003-4649-6366
Eder M
Edge M
Edgecombe G https://orcid.org/0000-0002-9591-8011
Edwards M
Ehses J
Eidels A
El-Ahmady SH
Elejalde E https://orcid.org/0000-0002-4755-1606
Elgendi M https://orcid.org/0000-0003-1831-0202
Elias G
Elitzur A
Emara S
Emery Thompson M https://orcid.org/0000-0003-2451-6397
Enderle R
Engesser S https://orcid.org/0000-0003-0425-2179
Eshraghian J
Espinal-Enríquez J
Esposito G https://orcid.org/0000-0002-9442-0254
Estellé P
Esteves da Silva JC
Etchegaray C
Everett C https://orcid.org/0000-0002-5889-3935
Exadaktylos G
Ezzeddine Z
Facchiano A
Fakhrullin R https://orcid.org/0000-0003-2015-7649
Falcone E https://orcid.org/0000-0002-9401-1354
Fan G
Fan W
Fandakova Y
Fang B
Fang C https://orcid.org/0000-0003-0915-7453
Fantuzzi N https://orcid.org/0000-0002-8406-4882
Farè S
Faria R http://orcid.org/0000-0001-9337-4324
Farina S
Farji-Brener A
Fathy ME
Fatouros N https://orcid.org/0000-0003-0841-3144
Faust K
Feilich K https://orcid.org/0000-0001-6057-897X
Feipeng J
Felsen G
Feng R
Fenton B
Ferguson C https://orcid.org/0000-0003-0986-7519
Fernandes I
Fernie A
Ferrari M
Ferreira F
Ferro V
Fiehler K
Figueira R
Filion G
Filipiak M https://orcid.org/0000-0001-7438-4885
Fimmel E
Findlay K
Fiorelli L
Fischer A
Fischer J https://orcid.org/0000-0002-5807-0074
Fischhoff B
Fleck C
Fleischmann P
Fleming S
Flessati L
Foffa D
Fokkens L
Fomine S
Font-Clos F
Foong J
Forbes-Inskip N https://orcid.org/0000-0002-7833-3521
Ford D
Forrester G https://orcid.org/0000-0001-8321-719X
Forstmeier W https://orcid.org/0000-0002-5984-8925
Fortelius M
Foth M https://orcid.org/0000-0001-9892-0208
Fournet M https://orcid.org/0000-0001-7875-1857
Fox G
Fraley RC
Frank M
Frank M https://orcid.org/0000-0001-9487-9359
Franklin S
Franz J https://orcid.org/0000-0001-9523-9708
Frasnelli E
Fry J
Fu R
Fuchs H
Fujihara T
Fullen M
Fürtbauer I https://orcid.org/0000-0003-1404-6280
Fuxjager M
Gale A
Galera-Laporta L
Galvan A
Galzitskaya O
Gangola S
Gao H https://orcid.org/0000-0001-6852-9435
Gao J https://orcid.org/0000-0002-3952-208X
Garcia-Esparza AT
Gårdfeldt K
Gårdmark A https://orcid.org/0000-0003-1803-0622
Gardner P
Garg H
Garnier S https://orcid.org/0000-0002-3886-3974
Gastaldo R
Gaston K
Gates I
Gates T
Gauden P
Ge J
Geitner N
Gemma S
Genner M
George P https://orcid.org/0000-0002-1080-098X
Georgiev Y
Georgopanos P
Georrge JJ
Gerisch A
Gernsbacher MA
Geso M
Gewirtz J
Ghouri N
Gijsen F
Gilani Z
Gillich GR
Gimenez O https://orcid.org/0000-0001-7001-5142
Giomi F https://orcid.org/0000-0002-7624-9457
Girard-Buttoz C https://orcid.org/0000-0003-1742-4400
Girault F
Gizynski K
Gizzi A
Gjini E https://orcid.org/0000-0002-0493-3102
Glauche I https://orcid.org/0000-0002-2524-1199
Glazier D https://orcid.org/0000-0001-7164-1823
Gluenz E https://orcid.org/0000-0003-4346-8896
Godyń K
Goel P
Goeppner S
Goicoechea H https://orcid.org/0000-0002-2092-7570
Goldenberg J
Gomes S
Gómez C
Gómez-Cifuentes A
Gondle RK
Gong B https://orcid.org/0000-0002-9464-3423
Gong F
Goodwin RA
Gopalaswamy A https://orcid.org/0000-0003-3841-3663
Goswami S
Goulet T
Gour N
Grabowski A
Graczyk-Zajac M
Gray K https://orcid.org/0000-0002-6071-4588
Green A
Green B
Greenfield A
Greenfield M
Gregertsen E
Grima R https://orcid.org/0000-0002-1266-8169
Grimes D
Gronenberg W
Grotheer M
Grunden A
Guan L
Gueriau P https://orcid.org/0000-0002-7529-3456
Gungoren C
Guo G https://orcid.org/0000-0003-1993-1633
Guo L
Guo W-y https://orcid.org/0000-0003-3589-4537
Guo Y
Gupta A
Gupta R
Gupta SK
Gupta S
Gupta S
Gupta VK
Gurau L
Gutt J
Guyader J
Haddad A
Haeri H https://orcid.org/0000-0002-5002-1497
Hagan K
Hahn C
Halbeisen G
Halley J
Halliday T https://orcid.org/0000-0002-4077-732X
Hämäläinen L
Han B
Han J
Han J
Han PKJ https://orcid.org/0000-0003-0165-1940
Han W-S
Han Y
Hanafi-Bojd AA
Handford M
Hansen I https://orcid.org/0000-0002-6560-2606
Harasti D https://orcid.org/0000-0002-2851-9838
Hardwicke T https://orcid.org/0000-0001-9485-4952
Harms K
Harned A https://orcid.org/0000-0002-6782-6224
Harper E
Harrington K https://orcid.org/0000-0002-7237-1973
Hartshorne J
Hartup B
Hasdemir B https://orcid.org/0000-0002-1071-1127
Hasenjager M https://orcid.org/0000-0003-2193-4120
Hashiba K
Hashiya K
Hasler C https://orcid.org/0000-0003-3242-7415
Hassan S-U
Hauber M https://orcid.org/0000-0003-2014-4928
Hayakawa A
Hayes L https://orcid.org/0000-0003-0713-416X
Hayes S
He G
He J
Heath S http://orcid.org/0000-0002-9822-8091
Heatwole H
Heck PR
Heddam S https://orcid.org/0000-0002-8055-8463
Hedge C
Held L https://orcid.org/0000-0002-8686-5325
Helle S http://orcid.org/0000-0002-6216-3759
Hellmann J https://orcid.org/0000-0003-0479-3960
Helm R
Helms V
Hemery M
Henderson A https://orcid.org/0000-0003-4384-4791
Henderson D
Hendry A
Hendy S
Henriques R
Heraldy E
Herbert-Read J https://orcid.org/0000-0003-0243-4518
Herborn K https://orcid.org/0000-0002-5913-7912
Herle M
Hermanns RL https://orcid.org/0000-0001-5577-5004
Hervé A
Hess U
Heuschele J
Heyd D
Higham P http://orcid.org/0000-0001-6087-7224
Hilgard J http://orcid.org/0000-0002-7278-4698
Hills T
Hirata D
Hirayama R
Hirst R
Hitch M
Hoch T
Hocking D https://orcid.org/0000-0001-6848-1208
Hoeffler J
Hoelzel R https://orcid.org/0000-0002-7265-4180
Hofmeester T
Hoi H https://orcid.org/0000-0002-3038-9673
Hojamberdiev M
Holtzer M
Honey M
Hopcroft P http://orcid.org/0000-0003-3694-9181
Hopcroft R
Horn D
Horne M
Hornig N
Horrocks N
Horváth A
Horvath G https://orcid.org/0000-0002-9008-2411
Hosen MI
Hoshino Y
Hossain M
Hosseini SE
Hou D
Hou J
Hou L
How CW https://orcid.org/0000-0002-5910-6542
Howes C
Hsueh Y-P
Hu C-P https://orcid.org/0000-0002-7503-5131
Hu D https://orcid.org/0000-0002-0017-7303
Hu L
Hua X
Huang H
Huang Q
Huang Y
Huang Z-G https://orcid.org/0000-0001-9648-3067
Huber M
Hudgins E
Hudon J
Hueckel T
Hui HY
Husbands P
Huyvaert K
Hwang Y
Iital A
Ilg P
Imaizumi S
Injaian A https://orcid.org/0000-0003-4633-5343
Inoue T https://orcid.org/0000-0003-3289-4478
Iosilevskii G https://orcid.org/0000-0002-4114-3214
Isager P
Ishizaka S https://orcid.org/0000-0001-9866-5809
Ishwar Prasad S
Islam MR
Ismail KN
Isobe N
Iwaniuk A
Jackson C https://orcid.org/0000-0002-3918-1721
Jacquin L http://orcid.org/0000-0002-0521-6113
Jaffe K
Jahan S
Jain M
Jain S
Jezierski J
Jäntti H
Jara F
Jayasinghe I
Jayasinghe S
Jell P
Jenkins M
Jensen K
Jessopp M https://orcid.org/0000-0002-2692-3730
Jia L
Jiang C
Jiang N
Jiang Q
Jin Z
Jixiong Z
John R https://orcid.org/0000-0002-3827-7617
Jones A
Jones B https://orcid.org/0000-0001-7777-0220
Jones C
Jones K
Jones P
Jordan O
Joseph SD
Joshi N
Jozwik K
Jungwirth A
Jurado-Sanchez B
Kabir M
Kadono K
Kafle A
Kajackaite A
Kalaimannan E
Kalidindi SB
Kammerer C https://orcid.org/0000-0002-0596-623X
Kan I
Kang HE
Kang T
Kannan B
Kapitaniak M
Kaplan J
Kapnoula EC
Kappeler P https://orcid.org/0000-0002-4801-487X
Kappenstein C
Kara HE
Karimi F https://orcid.org/0000-0002-0037-2475
Karuppiah M
Kashif M
Kasumyan A
Katterfeld A
Katti H
Kaviyarasu K
Kawahara S
Kawamura G https://orcid.org/0000-0001-6585-7636
Kawasaki H http://orcid.org/0000-0003-2713-2057
Kaya Y https://orcid.org/0000-0003-1506-7913
Ke Q https://orcid.org/0000-0002-2945-5274
Keagy J https://orcid.org/0000-0003-1235-4837
Keesey I https://orcid.org/0000-0002-3339-7249
Keizer A
Kelber A https://orcid.org/0000-0003-3937-2808
Kelly D
Kelly J
Kemp J https://orcid.org/0000-0003-3732-4992
Kempe V
Kench P
Kendall C https://orcid.org/0000-0003-4429-4496
Kendig M https://orcid.org/0000-0003-3337-6402
Kendon A
Ker A
Kermorgant S
Khamassi M
Khan AU
Khan SA
Killick R
Kim H-W
Kimball R
Kinateder M https://orcid.org/0000-0002-1669-3900
Kingsley M
Kirby K
Kiriliouk A
Kirkilionis M
Kisdi É https://orcid.org/0000-0002-7096-4222
Kiselev K
Kiss I https://orcid.org/0000-0003-1473-6644
Kitade O
Kittakoop P
Kivell T
Kmita A
Knieling F
Koblmueller S
Kohandel M https://orcid.org/0000-0003-0667-7269
Kolega J
Kolokolnikov T https://orcid.org/0000-0002-7179-4325
Komarova N https://orcid.org/0000-0003-4876-0343
Konečná M
Kong A-N
Kong J-Q
Kooh MRR
Korn C
Koroleva A
Koslover L
Kotrschal A https://orcid.org/0000-0003-3473-1402
Kotze D
Kragel P
Krajewski M
Kramer R
Krauel J
Krawczyk J
Krcmar S
Kreft I
Kreiman J
Kreitz C
Kripakaran P
Krishna A https://orcid.org/0000-0003-3838-9210
Kristoufek L
Krott A
Ksibi M
Kubow K
Kujala M
Kuku G
Kulkarni R
Kumar Barui A
Kumar A
Kumar G
Kumar N
Kuppens T
Kurasaki M
Kurniawan N
Kurokawa S https://orcid.org/0000-0003-0560-6653
Labuz J
Lacroix L
LaDage L
Ladisch M
Lahondère C https://orcid.org/0000-0003-1949-7865
Laiou A
Lambert O https://orcid.org/0000-0003-0740-5791
Lampert K https://orcid.org/0000-0002-0383-6132
Lane CA
Lane N https://orcid.org/0000-0002-5433-3973
Lane S https://orcid.org/0000-0002-3797-3178
Langley DP https://orcid.org/0000-0002-9136-8585
Langley E https://orcid.org/0000-0001-8980-8206
Larsson D
Laszka A
Late D
Lazaro A
Lazo MJ
Lazzara G https://orcid.org/0000-0003-1953-5817
Le Mouel C https://orcid.org/0000-0002-5102-5780
Leal M https://orcid.org/0000-0002-7441-1991
Lecheval V https://orcid.org/0000-0001-6041-2718
Lee A
Lee D
Lee J-C
Lee M https://orcid.org/0000-0002-3905-0887
Lee SY https://orcid.org/0000-0001-9493-1436
Lee S
Legal J-B
Leggat B https://orcid.org/0000-0003-4148-2555
Lehnert S https://orcid.org/0000-0002-3569-8299
Lei Y
Leitch D
Lema S https://orcid.org/0000-0002-2564-8992
Lemoine X https://orcid.org/0000-0003-1250-716X
Lench H
Lenormand M https://orcid.org/0000-0001-6362-3473
Leo E
Leongómez JD https://orcid.org/0000-0002-0092-6298
Lerman K
Levri E
Lewis A
Lewis J
Leys S
Li B http://orcid.org/0000-0002-7971-0085
Li B
Li F-L
Li G
Li H
Li H
Li H
Li J
Li J
Li M
LI QI https://orcid.org/0000-0003-0679-4385
Li S
Li Y
Li Y https://orcid.org/0000-0003-0357-4520
Li Y
Li Y
Liang R
Liang S-D
Liang Y
Liao Q
Liao R-Z
Liao Z
Libby E
Lillywhite H https://orcid.org/0000-0002-6410-4832
Lim K
Lim K-i
Lin C-J
Lin H
Lin J
Lin Y
Lin Z
Lincopan N
Lind J https://orcid.org/0000-0002-4159-6926
Linden D
Lindner E
Lindon J
Liu A
Liu G
Liu J
Liu J
Liu R
Liu W
Liu X
Liu X
Liu X
Liu X https://orcid.org/0000-0002-4422-4108
Liu X
Liu X
Liu Y http://orcid.org/0000-0002-9258-7237
Liu Y https://orcid.org/0000-0003-1440-4287
Liu Y https://orcid.org/0000-0003-1676-4446
Liu Z
Liu Z https://orcid.org/0000-0001-5936-1618
Livingstone S
Lo Franco R
Lohning A
Long J
Longo Jr L
Lopez H
Lord SR
Louis WNY
Loveday C
Loverdo C
Lowe R
Loyn RH
Lu A
Lu J
Lu J
Lu X https://orcid.org/0000-0003-4968-9462
Lu Y
Lubinski D
Lucas J https://orcid.org/0000-0001-9661-7985
Lucas S
Luda MP
Lui J
Luo S-K
Luo X
Luo X
Luscher A
Lynch A
Ma D http://orcid.org/0000-0001-6877-457X
Ma D
Ma H
Ma J
Ma Y
MacDonald J
MacInnes J
Mack J
Mackay J
Maclaren O
Macleod M
Macleod N
Madden J https://orcid.org/0000-0002-0691-0967
Madduri R
Magnotti J http://orcid.org/0000-0003-2093-0603
Mahdevari S
Mahjouri-Samani M
Mahmood Q
Makowicz A
Malkowski P
Malod-Dognin N
Malonza D
Mamidipudi GK
Maminski M
Mammarella I
Mammeri Y
Manariotis I
Mander Ü
Manell H
Manfrino C
Manhas Verbi Pereira F https://orcid.org/0000-0002-8117-2108
Manica L
Manier M https://orcid.org/0000-0001-9114-8649
Manivannan M
Manowska A
Manser M
Mao X
Maouene J
Marhl M
Marini G https://orcid.org/0000-0001-9721-7211
Marletto C https://orcid.org/0000-0002-2690-4433
Márquez J
Marshall H https://orcid.org/0000-0003-2120-243X
Marshall J
Martín M
Martin Crespo T
Martin P
Martin P https://orcid.org/0000-0003-2611-1211
Martindale R
Martínez Ramirez S
Martínez-Barrera G
Martins PT
Martins R
Marx F https://orcid.org/0000-0002-1029-4001
Mashkour M
Massoud E
Matechou E
Mather E
Mathot K https://orcid.org/0000-0003-2021-1369
Mathur M
Matsuzaki J
Matute R
Maust-Mohl M https://orcid.org/0000-0002-5764-4451
Mavroeidis A
Mayer-Gall T
Mayo M
Mazzocchi M
McBride S https://orcid.org/0000-0001-5120-0115
McCluskey A http://orcid.org/0000-0001-7125-863X
McDermott C
McDermott S
McDonald D
McDonald K https://orcid.org/0000-0003-4501-8565
McDonald R https://orcid.org/0000-0002-6922-3195
McFarlane DA
Mchedlov-Petrossyan N
McIntosh A
McManus J
McMullan R http://orcid.org/0000-0003-2677-8016
McPhaden M
Meadows J
Medal J
Medvedovici AV
Meehan M
Mehler A
Mehta V
Mei K-C https://orcid.org/0000-0001-6045-2154
Meléndez A https://orcid.org/0000-0002-5166-1840
Méndez-Rojas M
Meng F
Mercado III E
Meredith Smith M
Merkies K
Metternich N
Meyerhoff H
Mi L
Miano Pastor AC
Michell R
Michilietto D
Miguel E
Miksis-Olds J
Mikusinski G
Miler K https://orcid.org/0000-0001-7684-0629
Militao T https://orcid.org/0000-0002-2862-1592
Miller M http://orcid.org/0000-0002-4949-6903
Miller R
Mills D
Milton G https://orcid.org/0000-0002-4000-3375
Minas G
Mischi M
Mishra R
Miskam M https://orcid.org/0000-0001-9757-0883
Mizumoto N https://orcid.org/0000-0002-6731-8684
Mo D-c
Mobin S
Mobini S
Modesto S
Mogulkoc Y
Mohan S
Moitra P
Mol L https://orcid.org/0000-0001-5272-3671
Molnar J
Momigliano Paolo
Monaghan P
Mondaca-Fernandez I
Monteiro L
Moon B
Morand-Ferron J https://orcid.org/0000-0001-5186-7710
More M
Morelli S https://orcid.org/0000-0001-7551-8427
Moreno-Monroy AI
Morgani S
Mos B https://orcid.org/0000-0003-3687-516X
Motta P
Moura A
Mourao PR
Muggeridge A
Mukherjee K
Müller K
Mulsow J
Munday P
Murray K
Murray MB
Murtaza S
Musa M
Musick J
Musolesi M
Mustansar Hussain C
Mustapha A https://orcid.org/0000-0002-9077-4995
Nager R
Naghizadeh A
Nah S-Y
Nakamura I http://orcid.org/0000-0002-1386-7266
Nardi M
Nareddy P
Natalello A
Nawroth C https://orcid.org/0000-0003-4582-4057
Neda Z
Nelson E https://orcid.org/0000-0002-3454-4378
Nesbitt S
Nestor A
Neuschulz EL http://orcid.org/0000-0001-7526-2580
Neves RC
Newbury C
Nguyen DH
Nguyen PMV
Nguyen V
Niazi I
Nichols H https://orcid.org/0000-0002-4455-6065
Nie B
Niemiller M
Nijhof A https://orcid.org/0000-0002-4153-1284
Nikolaivits E https://orcid.org/0000-0002-8022-9272
Nilsonne G https://orcid.org/0000-0001-5273-0150
Ninaus M
Niu D
Noack B
Noble R https://orcid.org/0000-0002-8057-4252
Noè U
Nogaret A
Nord A https://orcid.org/0000-0001-6170-689X
Norfolk O
Norman D
Novas F
Nowicki J
Ntzoufras I
Nuijten M
Nyakatura J https://orcid.org/0000-0001-8088-8684
Nykanen M http://orcid.org/0000-0002-5489-7162
Nzioka F
Obot IB
Obracaj D
O'Brien K https://orcid.org/0000-0001-8972-9161
O'Dea R https://orcid.org/0000-0001-8177-5075
Ogawa M https://orcid.org/0000-0002-3781-2016
Ojha K
Okada D
Okada I
Okaiyeto K https://orcid.org/0000-0002-7211-714X
Okasha S https://orcid.org/0000-0001-6595-7557
Okuyama Y
Oleson E
Oliva D
Oliveira PF
Omar M
Ong K
Ong SH
Ono T
Opatowski L
Orci KM
Orłowski G
Ortiz F
Orton D
Orwin P
Ostergren J
Ottenburghs J
Otter K
Ouyang J
Owens H https://orcid.org/0000-0003-0071-1745
Owings C
Ozbakkaloglu T
Ozupek S
Padilla D
Padilla-Gamino J http://orcid.org/0000-0003-0478-9953
Palci A https://orcid.org/0000-0002-9312-0559
Palka D
Pallotta A
Palme R
Palminteri S
Palsdottir A https://orcid.org/0000-0002-5290-6862
Pan C
Pan J
Pan L
Pan M
Pan Y
Panaousis M
Pang J https://orcid.org/0000-0001-6965-4166
Pang R
Papadopoulos N
Papastamatiou Y https://orcid.org/0000-0002-6091-6841
papastathopoulos Y
Pappada S
Pappalardo F
Pappalardo L
Paps J https://orcid.org/0000-0003-2636-6636
Paredes R
Parent M
Parker W
Pascual-Ezama D
Pashler H https://orcid.org/0000-0003-3644-4909
Paterson CGB https://orcid.org/0000-0001-9928-3511
Patil MS
Patten M https://orcid.org/0000-0001-9689-2645
Patterson B
Patterson R
Peer B http://orcid.org/0000-0002-4423-0508
Peeters E
Pegg J
Pei W
Pęksa A
Pellicer-Rubio M
Peñalver E
Pendar H
Peng J
Pennock C
Pepperberg I https://orcid.org/0000-0001-9486-7323
Pereira R
Perez L
Pérez MA
Pérez-Rodríguez A
Perner J
Perpétuo I
Peter C
Petering D
Photopoulou T https://orcid.org/0000-0001-9616-9940
Pickering K
Piepenbrink K
Pierce D
Pilli NR
Pinheiro F
Pinheiro F
Pinto D
Pinzari F
Pirotta E https://orcid.org/0000-0003-3541-3676
Pisanski K https://orcid.org/0000-0003-0992-2477
Pisor A
Pitzer V
Podwyszynska M
Poh K
Polakof S
Poletto C https://orcid.org/0000-0002-4051-1716
Pomiankowski A https://orcid.org/0000-0002-5171-8755
Pongracz P https://orcid.org/0000-0001-5126-299X
Ponirakis D
Poologasundarampillai G https://orcid.org/0000-0002-8498-323X
Poropat SF
Porter M
Posa D
Pot G
Potvin D http://orcid.org/0000-0001-9296-9297
Pousa C
Powell L
Pozo OJ
Prabakaran S https://orcid.org/0000-0002-6527-1085
Prakash RV
Pramod RRT
Prasad V
Pratt B
Pratte Z
Preisig B
Pressman A https://orcid.org/0000-0003-0849-620X
PREVOST A https://orcid.org/0000-0002-4588-3316
Pripdeevech P
Prokopenko M https://orcid.org/0000-0002-4215-0344
Proppe DS
Przybylski A http://orcid.org/0000-0001-5547-2185
Psifidi A
Pugliese A
Pugnale A
Pugnaloni LA
Pumain D https://orcid.org/0000-0002-0730-4563
Putland R
Pyenson N
Qiao X https://orcid.org/0000-0002-8771-7877
Qing W
Quesque F
Quevedo Camargo C https://orcid.org/0000-0002-2947-765X
Quick N
Quintana M https://orcid.org/0000-0002-7036-8658
Qutachi O https://orcid.org/0000-0002-5026-7358
Rackow P
Radford C
Radoev B
Radwan E https://orcid.org/0000-0002-2784-3134
Raghupathi WRP
Rahman OU
Raia P https://orcid.org/0000-0002-4593-8006
Rainieri C
Raja MAZ
Ralph J
Ramachandran M https://orcid.org/0000-0002-5941-0536
Ramakrishna K
Ramos M
Ranagalage M
Rapposelli S https://orcid.org/0000-0003-0146-6358
Rasmussen A
Rathnaweera T
Rattray M
Ravikumar N
Rawlence N https://orcid.org/0000-0001-6968-6643
Raymundo L
Razo-Hernández RS
Read A
Realmuto J
Reber S https://orcid.org/0000-0001-8015-2538
Recio G
Reichard U
Reichert M https://orcid.org/0000-0002-0159-4387
Reimer J
Reisz R https://orcid.org/0000-0002-7454-1649
Rennison D
Resmini M https://orcid.org/0000-0001-8109-5352
Restif O https://orcid.org/0000-0001-9158-853X
Rezende CA
Rezk MR
Riascos J http://orcid.org/0000-0001-8631-8877
Ribeiro H https://orcid.org/0000-0002-9532-5195
Rivera-Perez J
Robert D
Roberts G https://orcid.org/0000-0002-6662-6829
Robertson C
Robinson E https://orcid.org/0000-0003-4914-9327
Roch M
Rodrigo A
Rodriguez Tovar F
Rodriguez-Cabal M
Rodriguez-Couto S
Roh C https://orcid.org/0000-0002-5681-0040
Rohner N
Rojas S
Rolando MA
Roldan A
Romão PR
Römer D
Ronchetti E
Rosa I
Rosati L
Rosen D
Rosenkranz A
Ross A
Ross C https://orcid.org/0000-0002-0067-4799
Rosseau R
Rothschild B
Rousselet G https://orcid.org/0000-0003-0006-8729
Roux V
Rubio-Fernandez P
Rufini A
Ruggera R
Rumeu B
Rungi A
Russo T
Ruthstein S
Ryan EJ
Rybski D
Ryu S
Sabareesh V
Sabbah S
Saberioon M
Sacchetti P
Safari M
Sahiner N
Sahoo B
Sakata S
Sakiyama T https://orcid.org/0000-0002-2687-7228
Sala J
Salameh C
Salanova Grau J
Salat H
Saleh T https://orcid.org/0000-0002-3037-5159
Salerno M
Sales L
Salter P
Sample D
Samuel S https://orcid.org/0000-0001-7776-7427
Samy A
Santacreu D
Sargis E https://orcid.org/0000-0003-0424-3803
Sarikaya MA
Sarkar S https://orcid.org/0000-0002-2037-0979
Sasmito AP
Sastry N
Säterberg T https://orcid.org/0000-0002-5881-7983
Savage P https://orcid.org/0000-0001-6996-7496
Saxton T
Scanlan P https://orcid.org/0000-0002-6733-9653
Scapini F http://orcid.org/0000-0001-7229-9572
Scatena R
Schalkwyk L
Scharf I https://orcid.org/0000-0002-8506-7161
Schieber J
Schild C https://orcid.org/0000-0002-6668-5773
Schlaghecken F
Schlupp I https://orcid.org/0000-0002-2460-5667
Schmidt B https://orcid.org/0000-0002-4023-1001
Schmidt J https://orcid.org/0000-0001-8549-0587
Schmitz B
Schmitz L https://orcid.org/0000-0003-0210-4383
Schneck A https://orcid.org/0000-0001-7035-0346
Schoepf V https://orcid.org/0000-0002-9467-1088
Schöneich S
Schooler J
Schooler J
Schultze HP
Schwartz JJ
Schwenk K https://orcid.org/0000-0002-0767-3940
Scrivner O
Scullion J
Searcy W
Seethapathi N
Segre P https://orcid.org/0000-0002-2396-2670
Segura A https://orcid.org/0000-0002-1989-8899
Seiler T-B https://orcid.org/0000-0001-8127-510X
Senn S
Seresinhe C https://orcid.org/0000-0001-6599-1325
Serrano JR
Serranti S
Shabbir J
Shahin G
Shakiba D
Shalev I
Shan Y
Shao W
Shapiro J
Sharot T
Sheehy E https://orcid.org/0000-0001-8749-2893
Sheffield CS
Shen T
Shen Y
Sheppard P
Sherbakov D
Shi J-W
Shi W
Shi Z
Shigenaka G
Shinkai Y
Shiozawa S
Siegert W https://orcid.org/0000-0001-7823-0293
Siegfried K
Sierksma J
Sigaki H
Sikiric M
Silbiger N https://orcid.org/0000-0003-4916-3217
Sillero-Zubiri C
Sills J
Silva P
Silva-Soares N
Silvina M
Simonis J
Singh Dr T
Singh U
Skagenholt M https://orcid.org/0000-0001-8554-1858
Skelton A
Sliney D
Slooten E
Sloutsky V
Smith D
Smith F http://orcid.org/0000-0003-4850-995X
Smith M https://orcid.org/0000-0001-5660-1727
Snowdon C
Sohail M
Sojo V
Solow A http://orcid.org/0000-0003-1978-1372
Song C
Song G
Song J
Song K
Song W
Songsasen N
Sorensen M https://orcid.org/0000-0002-5556-0502
Sparks A https://orcid.org/0000-0001-9916-2074
Spiegelhalter D
Spingler B
Špinka M
Spitoni G
Srinivas G
Srinivasa Rao S https://orcid.org/0000-0002-1308-7067
Srivathsa A
Stage H
Stairmand J
Stankovic M
Stanley C
Stansby P https://orcid.org/0000-0002-3552-0810
Statham M
Stavropoulou M
Steffen J https://orcid.org/0000-0002-0106-2452
Stegbauer L
Sterelny K https://orcid.org/0000-0003-3159-6698
Stervander M
Stevens AJ
Stevens C
Stewart F
Stewart H
Stien A https://orcid.org/0000-0001-8046-7337
Stigliani J-L
Stojanovic D https://orcid.org/0000-0002-1176-3244
Stolle D
Stout A https://orcid.org/0000-0001-7902-2509
Straley J
Street S https://orcid.org/0000-0001-8939-8016
Strobl-Mazzulla PH
Strouboulis J
Strozik G https://orcid.org/0000-0002-2927-4529
Sturdy C
Su H
Su N
Suen J https://orcid.org/0000-0002-9803-5900
Sues H-D
Sui N https://orcid.org/0000-0003-0411-0162
Sun Dr A
Sun H
Sun KW
Sun M
Sun Z
Suzuki S
Suzuki T https://orcid.org/0000-0001-6405-7653
Svardal H
Swaisgood R
Sweeney J
Szabo B https://orcid.org/0000-0002-3226-8621
Szczygielski T https://orcid.org/0000-0001-5108-8493
Szell M
Tacchella A
Tagliazucchi E https://orcid.org/0000-0003-0421-9993
Takagi D https://orcid.org/0000-0002-9738-1414
Talbot C https://orcid.org/0000-0003-4604-6891
Talhelm T
Tallet C
Tamayo A
Tambouratzis T
Tan A https://orcid.org/0000-0002-9227-4644
Tang L
Tang R
Tang Z
Tanimoto J
Tantos A
Taraba B
Tassetti AN
Tatone D https://orcid.org/0000-0001-6694-2656
Taulant M
Taylor Z
Teichroeb J
ten Cate C
Tentelier C
Teodoriu C
ter Haar S
Terna P https://orcid.org/0000-0002-7416-0870
Tersariol I
Thiébot J https://orcid.org/0000-0002-0038-1064
Thomas A
Thomas S
Thompson D
Thompson P https://orcid.org/0000-0002-5278-9045
Thompson R
Thorodden S
Thuy B
Tian H
Tian Z
Tietze DTh
Timmins L https://orcid.org/0000-0002-8707-8120
Timofeeva M
Tinghitella R https://orcid.org/0000-0002-0049-5539
Tinio P
Tizzoni M https://orcid.org/0000-0001-7246-2341
Tkaczynski P https://orcid.org/0000-0003-3207-2132
Tobalske B
Todd B
Todorov A
Tofanica B
Tokudome Y
Tönsing C
Toth L https://orcid.org/0000-0002-2568-802X
Tougaard J https://orcid.org/0000-0002-4422-7800
Travençolo B https://orcid.org/0000-0001-7690-301X
Trezise K
Trinder J
Trippier P
Trontelj J
Tryjanowski P https://orcid.org/0000-0002-8358-0797
Tsang D
Tseng J https://orcid.org/0000-0001-5335-4230
Tubiana L
Tunnicliffe V
Tupper P
Turner J https://orcid.org/0000-0003-2457-6138
Turner M
Turney S
Tuttle J
Twomey K
Tyson G
Tzamali E https://orcid.org/0000-0002-1756-7762
Ullmann CV
Uner O
Ungerfeld R
Unver A
Uomini N https://orcid.org/0000-0002-9898-6415
Vijaya Kumar V
Vallortigara G https://orcid.org/0000-0001-8192-9062
van Aert R
Van Cise AM
van de Lagemaat L https://orcid.org/0000-0002-2947-1557
Van Deelen T https://orcid.org/0000-0001-9471-6728
van den Akker O
van Loon K
Van Meeteren M
van Oostendorp M
Van Wallendael A
van Waterschoot T
Varga T https://orcid.org/0000-0002-5492-866X
Vargas-Bernal R https://orcid.org/0000-0003-4865-4575
Varma I
Vassileva E
Vasta M
Vazquez N
Vedenina VY
Velicer G
Veling H
Vella D https://orcid.org/0000-0003-1341-8863
Verhoef T
Victoria F https://orcid.org/0000-0002-8580-1813
Vidal-García M https://orcid.org/0000-0001-7617-7329
Videler J https://orcid.org/0000-0003-1226-9528
Vincenzetti S
Vipindas PV
Virdee B
Vitale P
Vlachos A
Vogel E
von der Haar T https://orcid.org/0000-0002-6031-9254
von Rueden C https://orcid.org/0000-0002-3225-5791
Vrtilek M
Vu T
Wagner G
Wai J
Wallenbeck A
Wan Ismail WN
Wang B
Wang C https://orcid.org/0000-0002-8669-1767
Wang D https://orcid.org/0000-0003-2477-4249
Wang D
Wang F
Wang F
Wang H
Wang H
Wang H-m
Wang J https://orcid.org/0000-0003-1326-4470
Wang L
Wang Q
Wang T-Y http://orcid.org/0000-0002-0793-1006
Wang T
Wang W
Wang X-y
Wang X
Wang Y
Wang Y
Wang Y
Wang Z
Wang Z https://orcid.org/0000-0003-1918-6282
Ward D
Ward E http://orcid.org/0000-0002-2789-2753
Warén A
Washburn D
Watkins C https://orcid.org/0000-0002-1207-6331
Watson D
Watson S-A https://orcid.org/0000-0002-9818-7429
Weaver D
Webb J
Weese D
Weese JS
Wei J
Wei X
Weijerman M https://orcid.org/0000-0001-5990-7385
Weil E
Weir BS
Weissburg M
Wells J
Wen H https://orcid.org/0000-0002-2045-729X
Whaley P https://orcid.org/0000-0003-4021-0785
Whittaker R
Whysall K
Wiehle M
Wignall P
Wild S https://orcid.org/0000-0002-5904-0096
Wilke A
Wilkinson R https://orcid.org/0000-0001-7729-7023
Williamson M
Willis A
Wilson R
Wilson S https://orcid.org/0000-0002-6282-4772
Wilson S
Wilson S https://orcid.org/0000-0001-8125-5133
Wilts B https://orcid.org/0000-0002-2727-7128
Winberg S
Winney I
Winters A https://orcid.org/0000-0003-2840-6267
Wiratsudakul A
Witjes V
Wittek S
Wittenberg R
Wojczulanis-Jakubas K
Wolf S http://orcid.org/0000-0002-3747-8097
Wollbrant C
Wong K-H
Woodgate J
Woodruff S
Wotherspoon S
Wright N
Wright T https://orcid.org/0000-0003-2859-5360
Wu B
Wu G https://orcid.org/0000-0002-0025-812X
Wu J https://orcid.org/0000-0002-7726-6216
Wu Q
Wu Q
Wu S
Wu X
Wu Z
Xia C
Xiao B
Xiao Y
Xie X
Xie Y
Xiong B
Xiong J
Xiong Y
Xu H
Xu J
Xu L
Xu P
Xu T
Xu W
Xu Y
Xu Z
Xue J
Yamamoto Y
Yampolsky L
Yan J
Yang B
Yang C
Yang D
Yang H
Yang H
Yang J https://orcid.org/0000-0001-5934-1537
Yang P
Yang W
Yang Y
Yang Z https://orcid.org/0000-0003-3351-7981
Yannis G
Yao B
Yao C
Yasseri T https://orcid.org/0000-0002-1800-6094
Yasunami M
Yave W
Ye Y
Yekutieli D
Yeo I-S http://orcid.org/0000-0002-6780-2601
Yesudass A
Yilmaz E
Yin W http://orcid.org/0000-0003-4411-1821
Ying Y https://orcid.org/0000-0002-3867-5893
Yokoyama T
Young L
Yousaf B
Yu B
Yu C
Yu L
Yu Q
Yu R
Yu Z
Yuvakkumar R https://orcid.org/0000-0001-6779-3453
Shilong Z
Zaadnoordijk L
Zamyatin A
Zarrouk A
Zeelenberg R
Zha L
Zhai S
Zhai S
Zhan G
Zhan S
Zhang B
Zhang C
Zhang C
Zhang G
Zhang G
Zhang J
Zhang J
Zhang L https://orcid.org/0000-0003-2431-869X
Zhang L
Zhang M
Zhang Q
Zhang Q
Zhang T-T
Zhang W
Zhang X
Zhang X https://orcid.org/0000-0002-0891-2560
Zhang Y
Zhang Y
Zhang Z
Zhao C-Y
Zhao L
Zhao S
Zhao Y
Zheng G
Zhong L http://orcid.org/0000-0002-8135-2766
Zhong X
Zhong X
Zhou H
Zhou S
Zhou S
Zhou T
Zhou W
Zhou W
Zhou X
Zhou Y
Zhou Z
Zhu C
Zhu C
Zhu G
Zhu J
Zhu J
Zhu M
Zhu M
Zhu X
Zhu Z-n https://orcid.org/0000-0001-6369-7552
Zhuravlev A http://orcid.org/0000-0003-1611-6916
Ziauddeen H
Zidan D
Zimmermann E
Zoellner S
Zoidis A
Zou L
Zou Q
Zuber A
Zuk NJ
Zuo J
Zwijnenburg M

